# Supplementary material for: Induction of Antimicrobial Protein S100A15 Expression by Oral Microbial Pathogens Is Toll-like Receptors-Dependent Activation of c-Jun-N-Terminal Kinase (JNK), p38, and NF-κB Pathways
Source: Int J Mol Sci. 2023 Mar 10;24(6):5348. doi: 10.3390/ijms24065348 (PMC10049289; doi:10.3390/ijms24065348)
Supplement: Supplementary file 1 [file ijms-24-05348-s001.zip › ijms-2256835-supplementary.pdf]

Material and Methods

Protein extraction and enzyme-linked immunosorbent assay (ELISA)

The total protein was extracted from GF and KB cells cultured under normal conditions in 150 mm Petri dishes for 24h before the stimulation with $2x10^4$ cells of either heat-inactivated *P. gingivalis* or *Sanguinis* bacteria. Forty-eight hours later, treated and control cells were subjected to the extraction of the total protein using 1ml of lysis buffer (150 mM NaCl; 50 mM Tris-HCl, pH 7.5; 1% Triton X-100; and 0.25% sodium deoxycholate). Then, the detection of S100A15 protein in both control and stimulated cells was performed by Sandwich enzyme immunoassay using an S100A15 ELISA kit (Cat#EKN48277, BioMatik corporation, USA) according to the manufacturer's protocol. Briefly, the microtiter plate provided in this kit has been pre-coated with an antibody specific to S100A15. 100µl (~5µg) of total protein extraction of control and stimulated cells were then added to the microtiter plate wells with a biotin-conjugated antibody specific to S100A15 protein. Next, Avidin conjugated to Horseradish Peroxidase (HRP) was added to each microplate well and incubated for 1h at RT with gentle continual shaking (~500 rpm). After the aspiration of the content, the wells were washed with 300µl of washing buffer 5 times. After washing had been done, 100µl of the detection antibody solution was added into each well and allowed to incubate for two hours at RT with gentle continual shaking (~500 rpm). At the end of the incubation, the contents were aspirated, and the wells were washed five times with >300 µL of Wash buffer per well. After the washing had been done, a 100 µL of 3,3,5,5-Tetramethylbenzidin (TMB) solution were added to each well. After the incubation of the plate for 30 minutes at RT, a 100µL of Stop solution was added to each well. 30 min following the addition of the stop solution, the absorbance of the reaction was made by ELISA reader at 450 nm.

Selection of the optimal concentration of anti-TLR2 and TLR4 antibodies. To determine the optimal concentration of either TLR2 or TLR4 that is essential to block the corresponding receptor. Both GF and KB cells were allowed to grow for 24h under normal conditions before the treatment with either IgG, TLR2, or TLR4 antibodies at different concentrations (1µg/ml, 5µg/ml, 10µg/ml, and 15µg/ml). Forty-eight hours, the total RNA of treated and control cells were subjected for the analysis of the basal expression of S100A15 by qRT-PCR as described in the section of material and methods in the main text of the manuscript.

Effect of the selected concentration of either anti-TLR2 or TLR4 on the cell viability.

To demonstrate whether the selected concentration of either anti-TLR2 and TLR4 antibodies, both GF and KB cells were allowed to grow for 24h under normal conditions. Then the cells were treated with 10µg/ml. Forty-eight hours post-treatment, the treated and control cells were subject to the analysis of the cell viability using MTT assay as described (Hassan et al., 2008).

Selection of the optimal concentration of the inhibitors of ASK1, JNK, and p38.

The selection of the optical concentration of the used inhibitors of ASK1, JNK, and p38 is based on our previous publications and the recommendation of the manufacturers as well. In this study, pilot experiments have been performed to confirm the optimal concentration of the inhibitors of ASK1 (Thioredoxin), JNK (SP60015), and p38 (SB-203580). The cell lines GF and KB were allowed to grow under normal conditions for 24h, then the cells were pretreated with a variable concentration of thioredoxin (0.5nM, 1nM, and 2nM), the inhibitor of ASK1 or with a variable concentration of SP600125, (10 µM, 20 µM, and 30 µM), the inhibitor of JNK or with a variable concentration of oSB-203580 (10 µM, 20 µM, and 30 µM), the inhibitor of p38 before the stimulation of with LPS (100 ng/ml). Forty-eight hours later, the total RNA of treated and control cells were extracted and subjected for the analysis of S100A15 expression by qRT-PCR as described in the section of material and methods of the main text.

Effect of the selected inhibitors of ASK1, JNK, and p38 on the cell viability of both GF and KB cells. To investigate whether the selected concentration of the inhibitors of ASK1, JNK1, and p38 influence the cell viability of either KB and KB cells, the cell lines GF and KB cells were allowed to grow for 24h under normal conditions before the pretreatment with a recommended concentration of either ASK1 inhibitor (1nM), JNK inhibitor (20µM) or inhibitor of p38 (20µM) and stimulation with LPS (100ng/ml) for regulated time intervals up to 72h. At the end of incubation, the cell viability was assessed. Treated and control cells were subjected to the analysis of cell viability using MTT assay as described (Hassan et al., 2008).P

Results

Detection of S100A15 by ELISA.

Next, we analyzed the induced protein expressions of S100A15 in both GF and KB cells after the stimulation of either heat-inactivated *P. gingivalis* or *Sanguinis* bacteria using ELISA. Although the observed basal expression of S100A15 is very weak to detect in control cells, the stimulation of either GF or KB cell was found to significantly induce the expression of S100A15 protein when compared to control cells ($p < .05$) as shown (Supl.1).

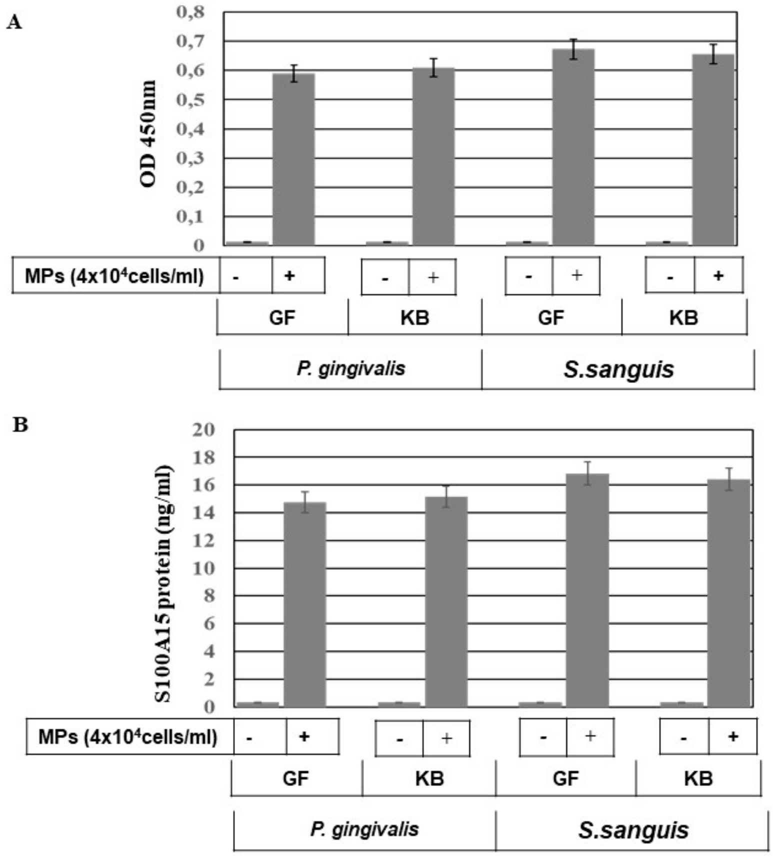

Figure S1. ELISA demonstrates the induction of S100A15 expression in both GF and KB cells following the stimulation with heat-inactivated *P. gingivalis* or *Sanguinis* bacteria. **A**) The absorbance value of the optical density (OD) of S100A15 protein in control and stimulated GF and KB cells. data are expressed as mean ± SD of six repeats in each experiment ($n = 3$). *$p < .05$. **B**) ELISA analysis of the S100A15 protein levels in stimulated and control GF and KB cells. All data are expressed as mean ± SD of six repeats in each experiment ($n = 3$). *$p < .05$.

Selection of the optimal concentration of anti-TLR2 and TLR4 antibodies.

To determine the optimal concentration of anti-TLR2 and TLR4 antibodies that are essential to block the function of toll-like receptors (TLR)-2 and 4. The treatment of both GF and KB cells with either IgG, anti-TLR2, or TLR4 antibodies with different concentrations (1µg/ml, 5µg/ml 10µg/ml, and 15µg/ml) for 48h. The treatment of the GF and KB cells with 1µg/ml or with 5µg/ml was found to partially inhibit the induced expression of S100A15 by either LPS or LTA in both cells. However, the observed inhibition of TLR2 or TLR4 increased thereafter to reach a maximum at a concentration 10µg/ml of either anti-TLR4 or TLR2 antibodies without showing any distinct increase in the noted inhibition at the concentration of 20µg/ml of the corresponding antibody as shown in Suppl. 2A, suggesting that the treatment of both GF and KB cells with 10µg of either anti-TLR2 or TLR4 antibodies is able to block LTA or LPS-induced expression of S100A15, respectively. As a consequence, we used a concentration of 10µg/ml of either anti-TLR2 or TLR4 antibodies to perform the inhibitory experiments in the present studies.

To investigate whether the selected concentration of anti-TLR2 and TLR4 antibodies influence the cell viability of either GF or KB cells. Both GF and KB cells were treated with 10µg/ml of either IgG or anti-TLR2 or TLR4 antibodies for regulated time intervals of up to 72h. Data obtained from the MTT assay (Suppl. 2B) indicate that the treatment of either GF or KB cells by either anti-TLR2 or TLR4 antibodies does not influence the cell viability of treated cells when compared with those of control one.

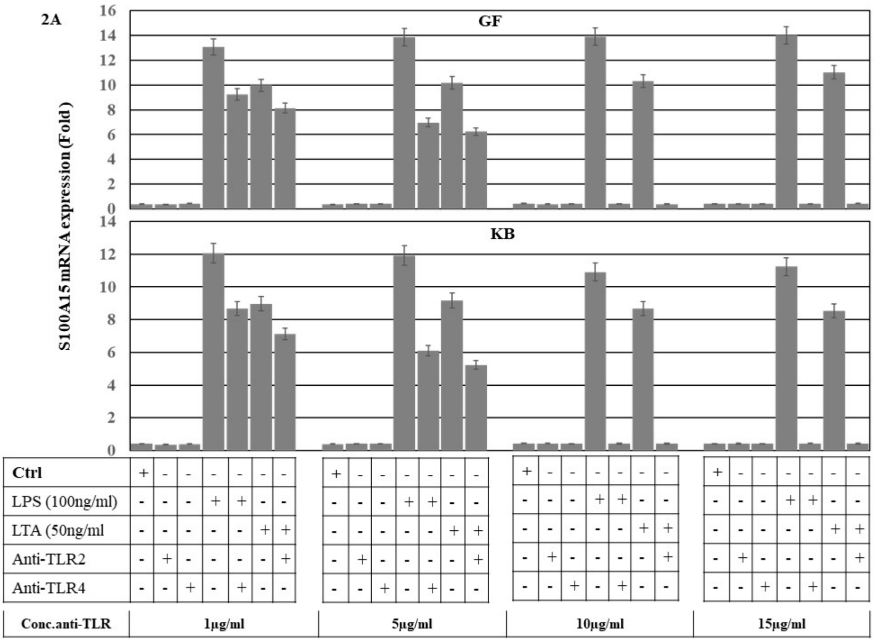

**Ctrl**	+	-	-	-	-	-	-	+	-	-	-	-	-	-	+	-	-	-	-	-	-	+	-	-	-	-	-	-
LPS (100ng/ml)	-	-	-	+	+	-	-	-	-	-	+	+	-	-	-	-	-	+	+	-	-	-	-	-	+	+	-	-
LTA (50ng/ml	-	-	-	-	-	+	+	-	-	-	-	-	+	+	-	-	-	-	-	+	+	-	-	-	-	-	+	+
Anti-TLR2	-	+	-	-	-	-	+	-	+	-	-	-	-	+	-	+	-	-	-	-	+	-	+	-	-	-	-	+
Anti-TLR4	-	-	+	-	+	-	-	-	-	+	-	+	-	-	-	-	+	-	+	-	-	-	-	+	-	+	-	-
**Conc.anti-TLR**	1µg/ml							5µg/ml							10µg/ml							15µg/ml						

.

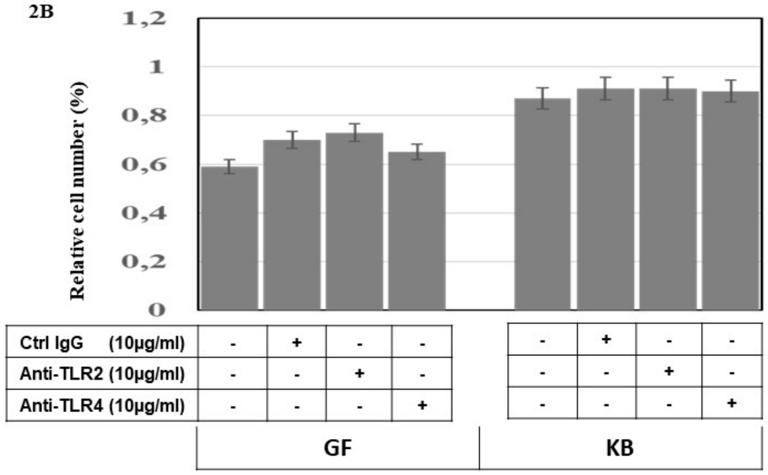

Figure S2. Selection of the optimal concentration of anti-TLR2 and TLR4 antibodies**. A**) qRT-PCR analysis of LPS/LTA-induced expression of S100A15 demonstrates the optimal concentration of anti-TLR2 and TLR4 antibodies that is essential to block LPS/LTA-induced expression of S100A15. Data are representative of three dependent experiments performed separately. **B**) MTT assay demonstrates that the selected concentration of either anti-TLR2 and TLR4 antibodies does not influence the cell viability of either GF or KB cells. Data are representative of three dependent experiments performed separately.

Selection of the optimal concentration of the inhibitors of ASK1, JNK, and p38

To investigate the optimal concentration of the inhibitors of ASK1, JNK, and p38, both GF and KB cells were pretreated with variable concentrations of the inhibitors of ASK1 (Thioredoxin), JNK (SP600125), and p38 (SB-203580) before the stimulation with either LPS or LTA for 48h. Then, the total RNA was extracted and subjected to the analysis of the expression of S100A15 by qRT-PCR. Data obtained from qRT-PCR analysis (Suppl. 3A) demonstrated a partial inhibition of LPS-induced expression of S100A15 following the treatment of the cells with 0.5nM of thioredoxin. The induced inhibition of S100A15 expression induced thereafter reached a maximum at the treatment with 1 nM of thioredoxin without showing any distinct increase following the treatment with 2nM of thioredoxin for the same time period of 48h when compared to control cells. These data indicate that the concentration of 1nM of thioredoxin is the optimal concentration to block the activity of ASK1. The analysis of both the inhibitory effect of Sp600125 (JNK inhibitor) and SB-203580 (inhibitor of p38) is shown in Suppl. 3B and 3C, respectively, suggested the optimal concentration of JNK inhibitor is 20µM, and the optimal concentration of the inhibitor of p38 is the concentration of 20 µM. In addition, the analysis of the cell viability of ether GF and KB cells following the pretreatment with an optimal concentration of the inhibitors of ASK1 (Fig. 4A), JNK (Fig. 4B), or p38 (Fig. 4C) does not influence the cell viability of either GF and KB cells.

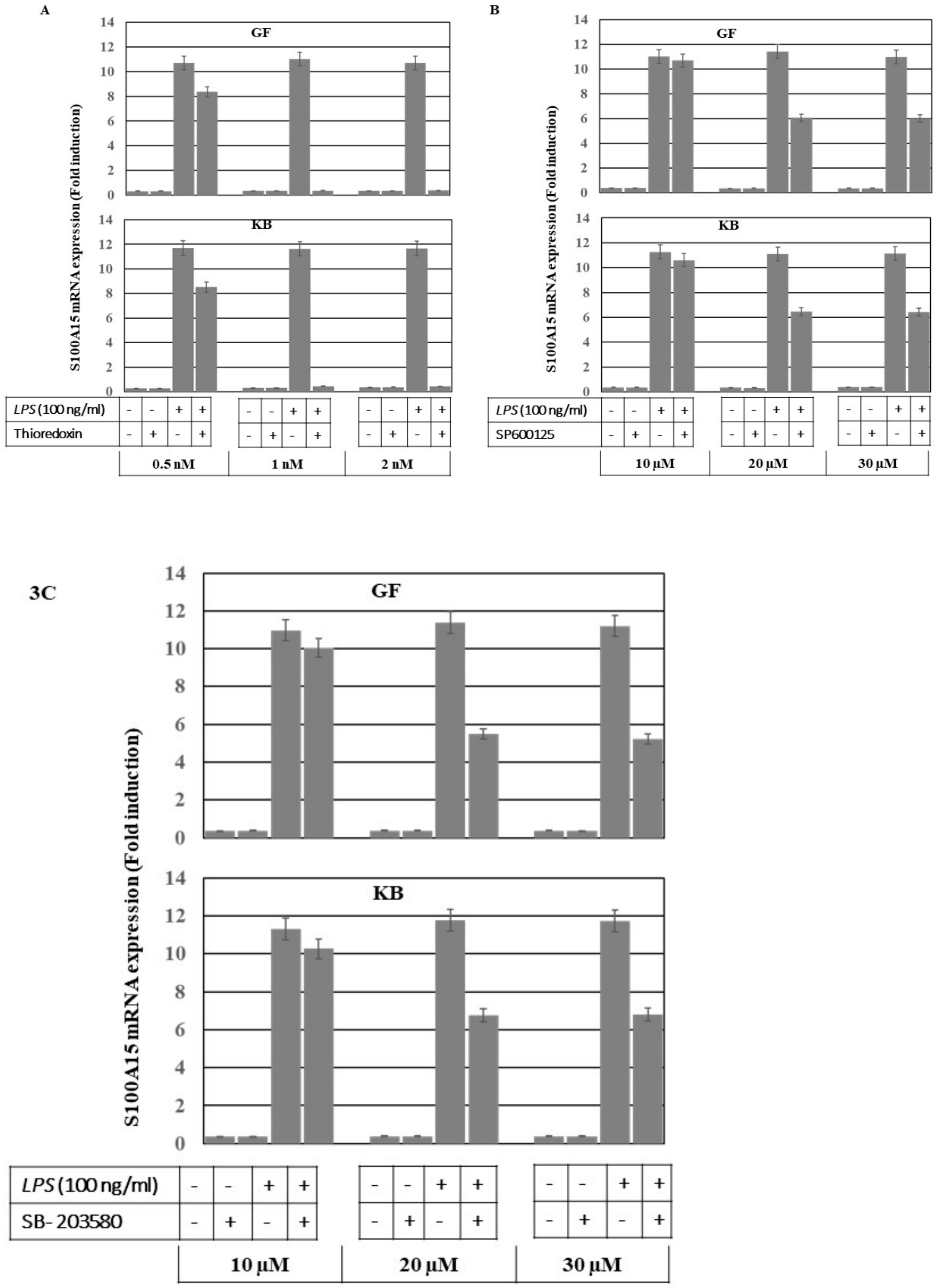

Figure S3. Selection of the optimal concentration of the inhibitors of ASK1, JNK, and p38. **A**) qRT-PCR demonstrates the optimal concentration of thioredoxin, the inhibitor of ASK1 that is able to inhibit the activity of ASK1 that is essential to mediate LPS-induced expression of S100A15. **B**) qRT-PCR demonstrates the optimal concentration of SP600125, the inhibitor of JNK that is able to inhibit the activity of JNK that contributes to the modulation of LPS-induced expression of S100A15. **C**) qRT-PCR demonstrates the optimal concentration of SB-203580, the inhibitor of p38 that able to inhibit the activity of p38 that is able to contribute to the modulation of LPS-induced expression of S100A15. Data are representative of three dependent experiments performed separately.

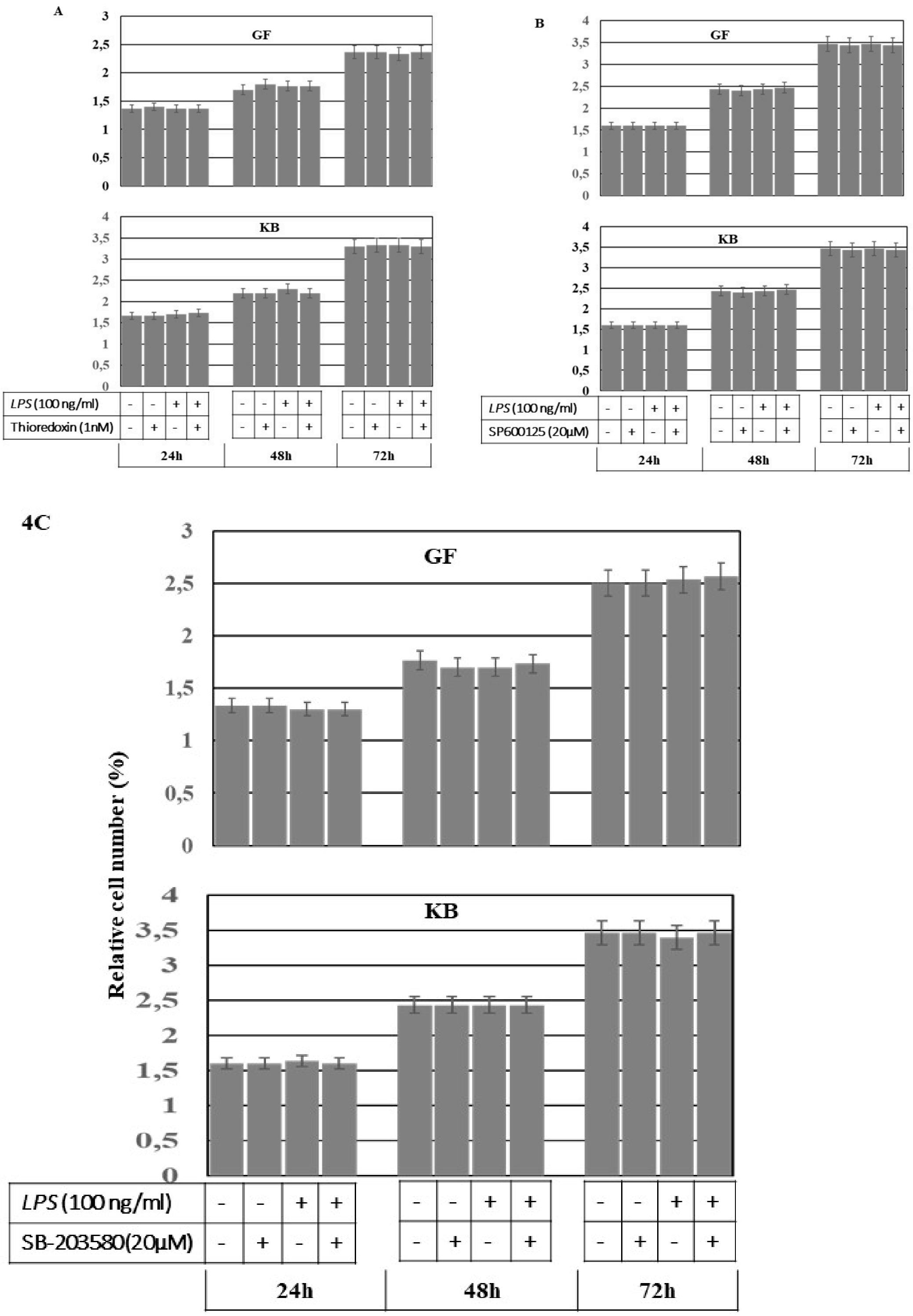

Figure S4. MTT assay demonstrates the effect of the selected inhibitors of ASK1 (**A**), JNK (**B**), and p38 (**C**) on the cell viability of GF and KB cells. Data are representative of three dependent experiments performed separately.

References

M. Hassan, A. Alaoui , O. Feyen, A Mirmohammadsadegh, F Essmann, A Tannapfel, E Gulbins, K Schulze-Osthoff, U R Hengge. The BH3-only member Noxa causes apoptosis in melanoma cells by multiple pathways. Oncogene 27 (33) (2008) 4557–68.
